# Pain Enhances the Auditory P2 but Attenuates Mismatch Response and P3a

**DOI:** 10.1002/ejp.70405

**Published:** 2026-10-09

**Authors:** Alexandre Lehmann, Marc Schönwiesner, Pierre Rainville

**Affiliations:** ^1^ Department of Otolaryngology Head & Neck Surgery McGill University Montreal Quebec Canada; ^2^ International Laboratory for Brain, Music and Sound Research (BRAMS) Montreal Quebec Canada; ^3^ Center for Research on Brain, Language and Music (CRBLM) Montreal Quebec Canada; ^4^ Department of Psychology University of Montreal Montreal Quebec Canada; ^5^ Centre de Recherche en Neuropsychologie et Cognition University of Montreal Montreal Quebec Canada; ^6^ Institute of Biology Leipzig University Leipzig Germany; ^7^ Département de Stomatologie Université de Montréal Montréal Quebec Canada; ^8^ Centre de Recherche de L'institut Universitaire de Gériatrie de Montréal Montréal Quebec Canada

**Keywords:** auditory processing, cognitive modulation, mismatch negativity (MMN), sensory salience, thermal stimulation, tonic pain

## Abstract

**Background:**

Auditory input is known to modulate the perception of pain, yet the reciprocal influence, how pain affects auditory processing, remains less well understood. We investigated how continuous tonic pain dynamically alters auditory cortical responses in humans.

**Methods:**

Using a three‐stimulus auditory oddball paradigm combined with EEG, we recorded event‐related potentials (ERPs) while participants experienced thermal stimulation under three conditions: baseline, non‐painful warmth, and pain. We examined multiple stages of auditory processing, including early sensory encoding (N1, P2), automatic deviance detection (MMN), and attentional reorienting (P3a).

**Results:**

Tonic pain modulated auditory ERPs in a stage‐ and component‐specific manner. Compared to baseline and warm conditions, pain was associated with enhanced P2 responses to distractors and reduced MMN and P3a amplitudes, although no statistically significant differences in behavioural performance were observed. This pattern suggests enhanced early salience detection alongside reduced pre‐attentive deviance detection and involuntary attentional shifts under pain. The warm condition selectively modulated N1 amplitude, indicating that some effects may be related to the affective and attentional demands of pain rather than somatosensory stimulation per se.

**Conclusions:**

Continuous tonic pain is associated with selective modulation of auditory processing, characterized by enhanced early sensory responsiveness and reduced higher‐order attentional engagement. This biphasic neural pattern, observed in the absence of measurable behavioural differences, is consistent with predictive coding and salience network accounts of pain‐attention interactions and highlights the sensitivity of ERPs for detecting pain‐related neural reweighting. The absence of statistically significant behavioural effects should be interpreted with caution and may reflect relatively subtle or context‐dependent influences of pain under the present task conditions.

**Significance Statement:**

This study is among the first to systematically examine how experimentally induced tonic pain modulates distinct stages of auditory processing in healthy individuals using high‐resolution ERPs, with a task design and hypothesis framework capable of teasing apart the cognitive mechanisms underlying these changes.

## 1 Introduction

Auditory perception arises from a hierarchical system integrating ascending and descending projections between the cochlea and cortex (Winer and Lee 2007). Top‐down circuits allow regulation of sensory input based on cognitive states like attention, expectation, and emotion (Guinan 2018). Within this framework, auditory processing is increasingly recognized as an active, context‐sensitive predictive process shaped by the continuous interplay between bottom‐up input and top‐down modulation, including pre‐attentive influences (Hołubowska and Schönwiesner 2025).

Pain, a salient signal that redirects attention, exemplifies top‐down modulation (Eccleston and Crombez 1999). Although auditory input (e.g., music) can modulate pain (Roy et al. 2008), less is known about the reverse; how pain alters auditory processing. Importantly, pain can act as a continuously present, internally salient signal, thereby exerting sustained top‐down influence on perceptual systems, including the processing of concurrent auditory input.

Electrophysiological approaches offer direct insight into how pain modulates sensory processing (Peyron et al. 2000). Pain is known to interfere with perception and attentional allocation, particularly under conditions of anticipation (Eccleston and Crombez 1999). Evidence from clinical populations further supports this interaction: chronic pain, such as low back pain, is linked to reduced attentional flexibility and diminished sensory filtering (Fan et al. 2018), whereas individuals experiencing episodic pain show habituation patterns more similar to healthy controls (Demirci and Savaş 2002). Together, these findings suggest that pain, particularly when sustained, can alter sensory processing through top‐down mechanisms affecting attentional control and sensory gating, effects that unfold across multiple stages of processing.

Event‐related potentials (ERPs), with high temporal resolution, are well‐suited for tracking pain‐related changes in auditory perception (Tivadar and Murray 2019). In particular, ERPs allow the dissociation of distinct stages of processing, from early encoding (N1, P2) and deviance detection (mismatch negativity–MMN) to attentional and evaluative responses (P3a, P3b) (Hofmann‐Shen et al. 2020), each potentially affected by pain.

Consistent with this framework, studies using ERPs have shown that pain can alter auditory processing (Torta et al. 2019). Chronic pain attenuates MMN and P3 responses to auditory deviants, alongside alterations in theta‐band phase coherence and cortical source activity (Castejón et al. 2024). Individuals with fibromyalgia also exhibit reduced right‐hemisphere MMN, suggesting impaired sensory gating and inhibitory control (Choi et al. 2015). These interactions may be mediated by shared thalamic relay systems, particularly the medial geniculate body and intralaminar nuclei, as hubs for nociceptive‐auditory‐attention integration (Suhnan et al. 2017). However, it remains unclear to what extent these alterations generalize to experimentally induced tonic pain in otherwise healthy individuals. These findings support the view that pain engages top‐down mechanisms that reshape auditory perception but also highlight the need to characterize such effects outside chronic pain populations.

In the present study, we examined how continuous tonic pain modulates auditory processing in healthy individuals. We used a thermal paradigm to induce background pain while participants completed an auditory oddball task involving noise stimuli. ERP recordings captured early and late neural responses, N1 and MMN (early), P2 and P3 (later), with a three‐stimulus oddball paradigm dissociating novelty (P3a) and task relevance (P3b). By comparing responses under painful and non‐painful conditions, we aimed to characterize pain‐related changes in auditory ERPs. According to the interruptive model of pain (Eccleston and Crombez 1999), we anticipated that tonic pain would preferentially impact higher‐level auditory processes associated with deviance detection and attentional orienting, potentially manifesting as reduced MMN and P3 components. At the same time, given evidence that pain can increase arousal and vigilance, we also considered the possibility that earlier sensory components such as N1 or P2 might be enhanced. These expectations motivated the present analyses, while allowing for component‐specific effects across multiple stages of auditory processing.

## 2 Materials and Methods

### 2.1 Participants

Twenty participants were recruited for the experiment and provided informed consent. Sample size was determined based on prior studies from our laboratory using similar auditory oddball and pain‐modulation paradigms. All participants had normal or corrected‐to‐normal vision and no history of hearing disorders or neurological conditions. The study procedures adhered to the Declaration of Helsinki and were approved by the local ethics committee (CÉRFAS‐2011‐12‐037‐A). One participant was excluded due to low EEG signal‐to‐noise ratio. The final sample included 19 participants (nine male), with a mean age of 25 years (SD = 3.3).

### 2.2 Stimuli

Three types of auditory stimuli were presented in pseudorandom order: standards (76% of trials), targets (12% of trials), and distractors (12% of trials). Stimuli were 60 ms in duration, with 5 ms rise and fall times shaped by a raised‐cosine gate, delivered at 70 dB SPL. The standard stimulus was the vowel /i/, spoken by a female speaker, with its pitch contour flattened to 220 Hz using the STRAIGHT pitch manipulation algorithm (Kawahara et al. 2001). The target stimulus was a pitch‐shifted version of the standard (+5%, F0 = 231 Hz), while the distractor stimulus was a white noise burst to be ignored. Auditory evoked potentials were recorded across sequential blocks using a three‐stimulus auditory oddball paradigm designed to ensure attention to auditory input and to reliably elicit oddball responses (Comerchero and Polich 1999). The 5% pitch difference between standard and target stimuli was calibrated in a pilot study to yield approximately 90% detection accuracy. Stimuli were presented with a fixed stimulus onset asynchrony (SOA; onset‐to‐onset interval) of 713 ms.

Thermal stimuli were delivered using a 3 × 3 cm contact thermode (TSA NeuroSensory Analyser, Medoc Ltd., Israel), applied to alternating sites on the right forearm. Two temperature levels were determined individually based on participants' pain sensitivity: a non‐painful warm temperature (mean = 41.9°C, SD = 1.2) and a moderately painful temperature (mean = 46.8°C, SD = 0.8). Each trial involved a 15‐s plateau at the target temperature, preceded by a baseline of 32°C. Temperature increased from baseline at a rate of 5°C/s and returned at a rate of 8°C/s. Inter‐stimulus intervals lasted 7.5 s. Participants were not informed that only two distinct temperatures were used.

### 2.3 EEG Recordings

EEG data were recorded using a BioSemi ActiveTwo system with 64 Ag/AgCl active electrodes embedded in a cap, positioned according to the extended 10–20 system. To monitor ocular artefacts, two external electrodes were placed at the outer canthi and one below the left eye to record electro‐oculogram activity. Additional electrodes were positioned on the nose and both mastoids. Active electrodes incorporate on‐site impedance transformation to minimize impedance‐dependent noise, eliminating the need for conventional impedance checks. Instead, electrode placement was adjusted to maintain direct current offsets near zero. EEG signals were acquired reference‐free at a sampling rate of 2048 Hz using the BioSemi ActiveTwo amplifier.

### 2.4 Procedure

Participants were seated comfortably in an electrically shielded, sound‐attenuated chamber, in a reclining armchair with head‐ and leg‐rests. They fixated on a central cross while completing the auditory task and receiving tonic thermal stimulation on the left forearm. Participants were instructed to attend to the sounds and to press a button with their right hand (using a response box, B‐Box, Tucker‐Davis Technologies) as quickly as possible upon detecting a target sound.

A pre‐experimental assessment was conducted during the same session to determine individualized thermal stimulation parameters. The thermal stimulator was manually applied to three alternating sites on the volar surface of the participant's right forearm. Using the ascending method of limits, thermal stimuli were applied to alternating sites on the right forearm, starting at 36°C and gradually increased until participants reported the first clear sensation of pain. Participants held a response box in their left hand and were instructed to press a button as soon as they experienced any pain sensation, defined as “a slightly burning, tingling, or pricking sensation.” Upon button press, the temperature rapidly returned to baseline at the pre‐determined rate. This procedure was repeated eight times, and the pain threshold was calculated as the average temperature of the last seven trials. Following threshold determination, participants were introduced to the visual analog scales (VAS) used to rate thermal sensation intensity and unpleasantness. They were familiarized with the on‐screen VAS and its operation using the response box. Participants were explicitly instructed on the structure of the intensity scale: values from 0 to 100 corresponded to non‐painful warm sensations, the transition to pain was defined at 101 (pain threshold), and values from 101 to 200 corresponded to increasing levels of heat pain. The unpleasantness scale (0–100) was presented separately and used to rate the aversive quality of the sensation. This scale was intentionally unipolar and did not include positive anchors, as it was designed to capture the degree of aversiveness rather than the full hedonic spectrum (i.e., pleasant vs. unpleasant). These VAS ranges were selected to ensure that warm and painful stimuli were perceptually distinct (moderate intensity), while avoiding extremes that could cause excessive discomfort or disengagement from the auditory task. Participants hen underwent a calibration phase consisting of 15‐s thermal stimulations with pseudorandom temperatures ranging from 39°C to 48°C. During this phase, they were introduced to the auditory oddball detection task and completed a brief (~5 min) practice session without thermal stimulation. Based on VAS ratings obtained during these stimulations, stimulus–response curves were generated for each participant. Two individualized target temperatures were then selected: one corresponding to a moderate warm sensation (VAS ~60/200 on the intensity scale) and one corresponding to a moderate pain sensation (VAS ~160/200). To refine these estimates temperatures were further adjusted in 0.5°C increments above and below the initially selected values. Final stimulation temperatures were determined based on this second calibration. Participants then completed one final training block of the auditory task to ensure task comprehension. Once confirmed, EEG cap preparation and electrode setup were initiated.

The auditory task was presented in five blocks, each corresponding to a single thermal condition (warm or painful) maintained throughout the block. Block order was pseudorandomized and counterbalanced across participants. To reduce anticipatory anxiety, conditions alternated in a predictable sequence between warm and pain. Each block lasted approximately 4.5 min and followed the structure illustrated in Figure 1. A visual cue signalled the start of the block, after which the auditory task began. During each block, participants received four 15‐s thermal stimulations interleaved with 15‐s baseline periods. This was followed by a 22‐s rating period during which no auditory or thermal stimulation was presented. For safety, participants had access to an alarm button throughout the experiment; pressing it terminated both thermal stimulation and the auditory task. No participants chose to interrupt the experiment.

**FIGURE 1:**
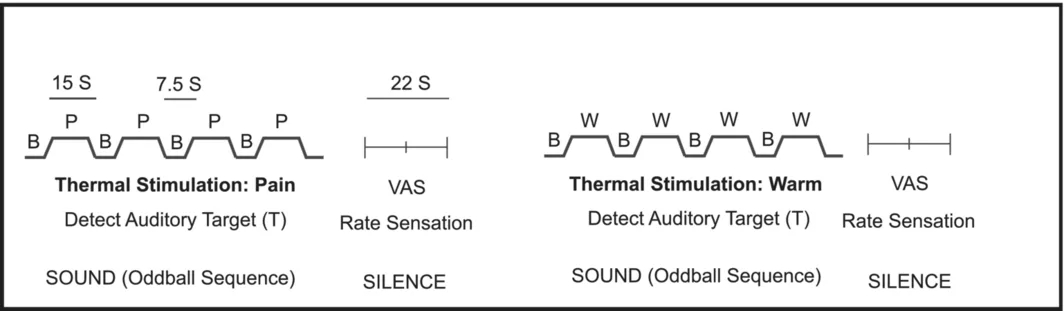
Schematic of the experimental block structure for pain (left) and warm (right) thermal stimulation conditions. Each block consisted of four 15‐s thermal stimulation periods (Pain [P] or Warm [W]), each separated by a 7.5‐s baseline (B) period at 32°C. During stimulation and baseline periods, participants performed an auditory oddball task involving detection of infrequent target tones (T) within a continuous stream of standard and distractor sounds. At the end of each block, there was a 22‐s silent period during which participants rated the intensity and unpleasantness of the preceding thermal stimulation using visual analog scales (VAS). B: Baseline; P: Pain; W: Warm; T: Target sound.

After each block, the experimenter entered the room to verify the ratings with the participant and repositioned the thermode to avoid local skin fatigue. During each rating period, participants evaluated the thermal stimulation experienced in the preceding block using VAS displayed on a computer screen. The VAS appeared horizontally, with the cursor initially positioned at a random location. The current value of the cursor was displayed above the scale in real time. Participants first had 11 s to rate the intensity of the thermal sensation on a scale from 0 (no sensation) to 200 (extremely painful), with 101 corresponding to the pain threshold (i.e., the slightest pain detectable). They then had an additional 11 s to rate the unpleasantness of the sensation on a scale from 0 (not unpleasant) to 100 (extremely unpleasant). As the unpleasantness scale was unipolar, ratings near zero were interpreted as reflecting the absence of unpleasantness rather than the presence of positive hedonic valence. All ratings were made using the same response box used during the auditory oddball task.

### 2.5 Analysis

For the auditory task, accuracy and mean reaction times were computed and averaged separately for each thermal condition (Pain, Warm, Baseline). To verify that the thermal stimulation elicited the intended perceptual experiences, mean ratings of intensity and unpleasantness were compared between the Pain and Warm conditions.

EEG data were resampled to 256 Hz, re‐referenced to the averaged mastoids, and band‐pass filtered using a zero‐phase Butterworth filter (0.01–50 Hz, 12 dB/octave). Data were visually inspected for artefacts, and consistently noisy channels were interpolated from surrounding electrodes. The continuous data were segmented into epochs ranging from −50 to 700 ms relative to stimulus onset. Trials exceeding ±100 μV or flagged by EEGLAB's automatic iterative rejection procedure were excluded (initial threshold set at 5 standard deviations; Delorme and Makeig 2004). Eye‐blink artefacts were identified and removed using independent component analysis (ICA). As is standard in P3 analyses, epochs containing incorrect responses or false alarms were excluded. In total, approximately 7% of trials were rejected. The mean number of accepted epochs per participant per thermal condition was 255 for standards, 40 for targets, and 42 for distractors. Baseline correction was applied using the −50 to 0 ms pre‐stimulus interval, and epochs were averaged separately by stimulus type and thermal condition. We investigated the impact of thermal condition on auditory ERPs elicited by the three‐stimulus oddball paradigm, focusing on N1 and P2 components across all stimuli, the P3a to distractors, the P3b to targets, and the MMN, computed as the target‐standard difference waveform (Comerchero and Polich 1999). Component latencies were estimated using peaks in the grand‐average global field power (Lehmann and Skrandies 1984).

To control for multiple comparisons and leverage the spatiotemporal structure of the EEG data, statistical analyses were conducted using a non‐parametric, cluster‐based permutation approach implemented in BESA Statistics (Maris and Oostenveld 2007). Statistical inference was based on cluster‐level effects spanning adjacent electrodes and time points, rather than on precise onset or peak latencies of individual samples, consistent with current methodological recommendations for cluster‐based permutation inference (Sassenhagen and Draschkow 2019). Channel neighbourhoods were defined using a spatial threshold of 4 cm, and statistical significance was assessed using a conservative threshold of *p* = 0.001.

A one‐way, permutation‐based ANOVA was conducted for each stimulus type (standard, target, distractor) with thermal condition (Baseline, Warm, Pain) as the within‐subject factor. Significant clusters were required to include at least four electrodes, exhibit effects sustained over a minimum of 20 ms, and overlap with the component‐specific time windows defined by the GFP analysis. The 20 ms minimum duration criterion reflects standard practice in ERP analysis, based on the temporal resolution of cortical potentials and the need to exclude brief fluctuations unlikely to represent reliable neural responses. When a significant main effect was observed, post hoc pairwise comparisons were performed using a permutation‐based Scheffé's test. Resulting pairwise clusters were necessarily subsets of the main ANOVA‐identified clusters, providing a hierarchical control over Type I error.

## 3 Results

### 3.1 Behavioural Measures

As expected, the painful thermal stimulation was rated as both painful and unpleasant, with a mean intensity rating of 145 (SD = 4.2) and a mean unpleasantness rating of 62 (SD = 2.9). In contrast, the warm stimulation was perceived as non‐painful and associated with low unpleasantness ratings (mean intensity: 42 (SD = 7.2); mean unpleasantness: 13 (SD = 3.6)). Participants consistently rated the warm condition as significantly less intense and less unpleasant than the pain condition (*t*(19) = 17.3 and *t*(19) = 11.9, respectively; *p* < 0.0001 in both cases). Participants demonstrated high overall performance on the auditory oddball task, with a mean target detection accuracy of 88% and a mean reaction time of 394 ms. No significant effect of thermal condition on task performance was observed: neither accuracy nor reaction time differed significantly across baseline, warm, and pain blocks (*p* > 0.15).

### 3.2 Auditory‐Evoked Potentials

Auditory stimulation reliably elicited well‐defined ERP components typical of the three‐stimulus oddball paradigm (N1, P2, MMN, P3a, and P3b; Figure 2). Cluster‐based permutation analyses revealed significant effects of thermal condition on multiple components, spanning both early sensory responses and later attentional processes. For standard sounds, a significant frontocentral cluster was observed within the N1 time range (*p* < 0.001; Figure 3, top left). Post hoc comparisons indicated greater N1 amplitude under Pain relative to Warm stimulation (*p* < 0.001). MMN‐related activity was attenuated during Pain relative to both Baseline and Warm conditions. This effect was reflected in a significant frontocentral cluster in the target‐standard difference waveform overlapping the MMN time range (*p* < 0.001; Figure 3, top right). Post hoc analyses confirmed lower MMN amplitude in the Pain condition relative to both Baseline and Warm (*p* < 0.001). Neural responses to task‐irrelevant distractor sounds were also modulated. Two significant clusters were identified (Figure 3, bottom row), corresponding to early (P2‐related) and later (P3a‐related) processing stages. The P2‐related cluster showed greater amplitude under Pain compared to Warm stimulation (*p* = 0.001), whereas the P3a‐related cluster showed reduced amplitude under Pain relative to both Baseline and Warm conditions (*p* < 0.001). No significant differences were observed between Baseline and Warm for either cluster.

**FIGURE 2:**
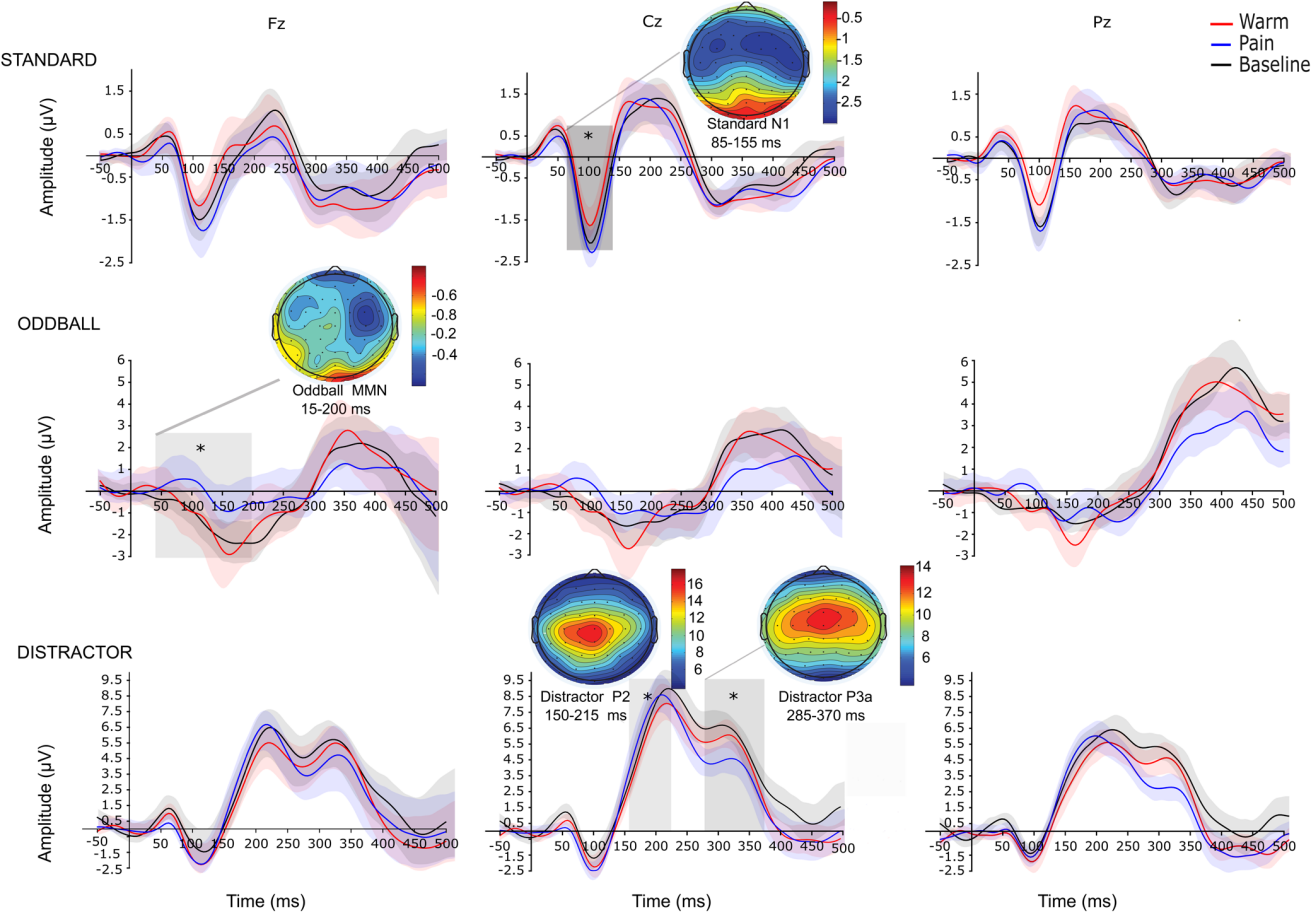
Grand‐average ERPs for standard, oddball (target), and distractor stimuli across the three thermal conditions: Baseline (black), Warm (red), and Pain (blue), recorded at three midline electrode sites (Fz, Cz, and Pz). Coloured shaded areas around the waveforms represent standard error of the mean (SEM); grey shaded areas indicate time windows with significant clusters identified by permutation‐based ANOVA (*p* = 0.001). Scalp maps illustrate the mean amplitude within the corresponding time windows for each significant ERP component.

**FIGURE 3:**
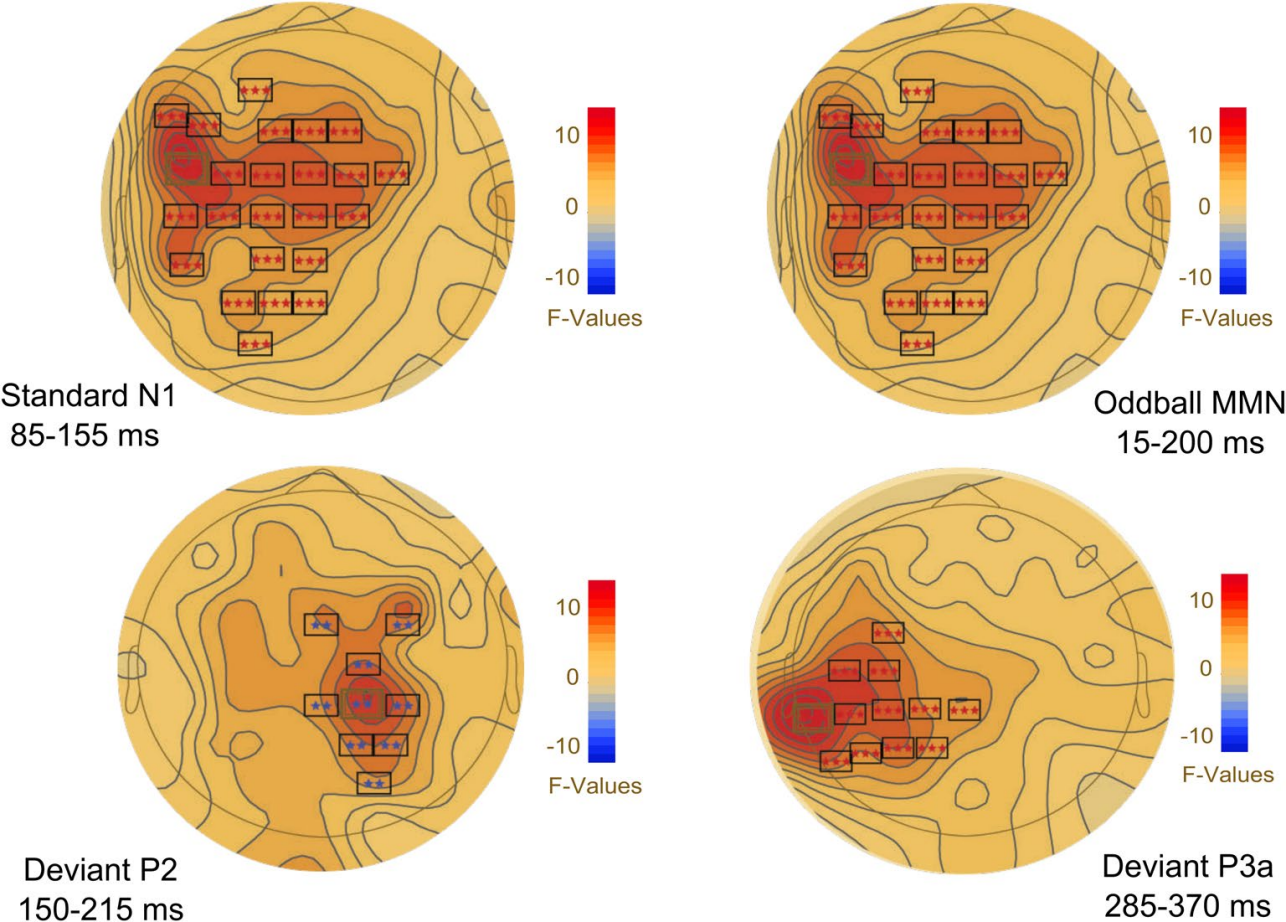
Topographical cluster maps showing statistically significant effects of thermal condition on ERP components, identified via cluster‐based permutation ANOVA (*p* = 0.001). Red boxes indicate spatial clusters where F‐values exceeded the threshold, and asterisks denote time–electrode combinations that survived multiple comparisons correction. Maps are shown for: Standard N1 (85–155 ms), Oddball MMN (150–200 ms), Deviant P2 (150–215 ms), and Deviant P3a (285–370 ms). Warm colours represent positive F‐values, with the strongest effects over frontocentral and centroparietal scalp regions. ***p < 0.001, **p = 0.001.

## 4 Discussion

This study examined how tonic pain influences auditory cortical processing during active listening, using a three‐stimulus oddball paradigm under baseline, non‐painful warm, and painful thermal conditions. Auditory stimuli reliably elicited canonical ERP components (N1, P2, P3a, P3b, and MMN), allowing for a detailed investigation of pain‐related modulation across multiple processing stages. Results showed that tonic pain altered auditory responses in a component‐specific manner, including enhancement of the early P2 response to distractors and attenuation of MMN and P3a amplitudes. No significant effects of thermal condition on behavioural performance were observed. Given the estimated sensitivity of the present design (Cohen's *d* = 0.67), these findings suggest that any potential behavioural effects of tonic pain, if present, are likely smaller in magnitude than the neural effects observed here. Notably, even warm stimulation was associated with modulation of N1 amplitudes, suggesting that thermal input, even at non‐painful levels, may influence early auditory processing, potentially depending on its salience and affective properties.

One of the central findings of this study was the attenuation of MMN during painful stimulation, consistent with altered automatic deviance detection under tonic pain (Näätänen et al. 2014). The MMN, a pre‐attentive index of auditory sensory memory and prediction error signalling (Näätänen et al. 2014), was reduced under pain relative to both baseline and warm conditions. This finding is consistent with results reported by Fan et al. (2018), who observed a similar MMN reduction during acute pain that was absent when pain was no longer present. Together with evidence from chronic pain patients showing MMN normalization following analgesic treatment, these results support the view that pain‐related MMN changes reflect a dynamic, state‐dependent modulation of pre‐attentive auditory processing rather than permanent structural alterations.

In our context, the suppression of MMN may reflect a dynamic reallocation of cognitive resources from auditory deviance monitoring toward processing somatic threat signals. This interpretation aligns with predictive coding frameworks, wherein precision‐weighted prediction errors are modulated by contextual salience (Heilbron and Chait 2018), as well as with models of the salience network that emphasize the prioritization of behaviourally relevant nociceptive input (Iannetti and Mouraux 2010; Legrain et al. 2011). Pain‐related effects on both N1 and MMN were most pronounced over frontocentral regions, suggesting the involvement of auditory and prefrontal areas linked to early sensory encoding and mismatch detection (Garrido et al. 2009; Kucyi et al. 2012).

Although no statistically significant modulation of P3b amplitude was observed during pain, these results should not be interpreted as evidence for the absence of an effect. Rather, they suggest that any potential modulation of P3b by tonic pain may be relatively subtle under the present task conditions. The P3b component reflects working memory updating and the allocation of attention to task‐relevant stimuli (Polich 2007) and is known to be sensitive to cognitive load (Wronka et al. 2012). In the context of the relatively low task demands of the present paradigm, pain‐related effects on P3b activity may have been limited or context‐dependent.

This interpretation is supported by Moore et al. (2017), who reported that thermal pain disrupted behavioural performance only under high‐load conditions (e.g., n‐back working memory) but had minimal effects on simpler tasks. At the neural level, Seminowicz and Davis (2007) found that pain‐related reductions in task‐related neural activity occurred mainly under low‐load conditions, suggesting that pain may differentially impact behavioural and neural systems depending on task demands. Additional support comes from Troche et al. (2015), who showed that both P3a and P3b amplitudes were reduced by acute pain during a distraction‐based task. These findings indicate that P3b modulation may emerge not only under high attentional load, but also in paradigms that involve frequent attentional reorienting, potentially increasing susceptibility to interference from pain.

Furthermore, Houlihan et al. (2004) observed increased frontal slow‐wave activity during pain regardless of memory load, even in the absence of behavioural deficits. Although this activity is distinct from the classic P3b, it might reflect compensatory prefrontal engagement or altered control processes that are not fully captured by behavioural measures. Taken together, these findings suggest that the absence of a statistically significant P3b modulation in the present study should be interpreted with caution. It remains possible that pain‐related effects on P3b are subtle and depend on task characteristics, such as cognitive load or attentional demands, which may influence the extent to which such effects can be detected.

To better understand how pain alters processing of novel, task‐irrelevant sounds, we examined ERP responses to distractor stimuli. Although the P3a follows the P2 temporally, findings are presented here in functional order, beginning with early salience encoding (P2) and moving to later attentional orienting (P3a), to reflect the hierarchical structure of novelty processing under pain. This pattern was characterized by an enhancement of the P2 component during pain, accompanied by a reduction in P3a amplitude. The P2 component, typically observed over centroparietal regions, is associated with early perceptual evaluation and salience detection (Luck and Hillyard 1994; Zouridakis et al. 1998). In the present study, P2 modulation under pain showed a more lateralized, parietal distribution, consistent with the engagement of salience and attention‐related networks, including the temporoparietal junction and ventral attention system (Downar et al. 2000; Corbetta and Shulman 2002). The enhancement of P2 during pain may reflect increased sensory vigilance or heightened responsiveness to novel input, consistent with the heightened arousal and unpleasantness ratings reported during the pain blocks. Similar increases in P2 amplitude have been reported in anxious individuals attending to threat‐related cues (Eldar et al. 2010), in response to attended pain stimuli (Chan et al. 2012) in patients with chronic pain syndromes such as fibromyalgia (e.g., Cardoso et al. 2024; Fernandes‐Magalhaes et al. 2024), and in healthy participants exposed to thermal pain under low cognitive load (Wang et al. 2021). Together, these findings are consistent with the interpretation that P2 enhancement may index an affectively modulated, bottom‐up amplification of salient or aversive sensory signals.

In contrast, the novelty P3a, a marker of involuntary attention shifts toward unexpected, task‐irrelevant stimuli, was reduced under pain, particularly over left temporoparietal regions. This reduction suggests that, although early salience processing was enhanced, subsequent attentional reorienting may have been diminished under tonic pain. This pattern is consistent with the “interruptive model” of pain (Eccleston and Crombez 1999), which proposes that nociceptive input captures central attentional resources and limits the processing of external stimuli. Similarly, Troche et al. (2015) reported reduced P3a and P3b amplitudes in a distraction paradigm involving acute pain, supporting the idea that pain can interfere with later‐stage attentional processing, particularly in tasks requiring attentional shifts or allocation. The observed reduction in P3a is also in line with diminished novelty responses reported during emotional arousal or stress (Keil et al. 2007), as well as with increased frontal slow‐wave activity during pain, even in the absence of measurable behavioural deficits (Houlihan et al. 2004), suggesting the presence of latent cognitive load. This interpretation is further supported by neuroimaging findings (Peyron et al. 2000), which show that nociceptive input engages a distributed network involving both sensory and frontoparietal regions, including the anterior cingulate cortex and prefrontal areas implicated in attentional control and salience processing. Together, these findings support the view that tonic pain may bias attentional resources toward internally salient, threat‐related input, potentially at the expense of monitoring external stimuli.

Moreover, these findings are consistent with a biphasic modulation pattern, characterized by an early amplification of perceptual encoding (P2) followed by suppression of automatic orienting (P3a) (Troche et al. 2015). This temporal organization may reflect a shift in processing priorities under tonic pain, whereby novel auditory stimuli are initially registered as salient but may be deprioritized during later evaluative stages, possibly to conserve cognitive resources for internal threat monitoring or pain regulation (Wang et al. 2020). Accordingly, neural modulations may occur even when overt task performance remains unchanged, reflecting a trade‐off between attending to external information and monitoring internally salient signals (Eccleston and Crombez 1999).

Importantly, the warm (non‐painful) condition was associated with relatively limited modulation of ERP components, with the exception of reduced N1 amplitude to standard stimuli compared to the pain condition. This effect may reflect generalized sensory gating or reduced auditory salience in the presence of concurrent somatosensory input (Spooner et al. 2020). Although no statistically significant effects of warm stimulation were detected on MMN, P2, or P3a components, this does not exclude the possibility of more subtle or distributed effects that may not have been detectable with the present design and sample size (Eccleston and Crombez 1999; Dick et al. 2006). Taken together, the overall pattern suggests that auditory ERP modulations observed in the present study are more pronounced under painful stimulation and may be related to the affective and attentional demands associated with pain, rather than to thermal stimulation alone (Fan et al. 2018; Spooner et al. 2020).

In sum, this study provides evidence that tonic pain is associated with temporally and spatially selective modulation of auditory cortical processing. Our results are consistent with a biphasic modulation pattern characterized by enhanced early encoding (P2) and suppressed later orienting (P3a), alongside reduced deviance detection (MMN). These findings suggest that sustained pain may dynamically influence how auditory information is filtered, evaluated, and responded to, potentially biasing processing toward internally salient nociceptive signals relative to external sensory input. This pattern is compatible with contemporary models emphasizing the interaction between top‐down attentional control and bottom‐up salience detection in pain‐attention interactions (Tracey and Mantyh 2007; Legrain et al. 2009). Moreover, the present data highlight the sensitivity of ERP measures for detecting neural modulations that may occur in the absence of measurable changes in overt behavioural performance. Future studies should examine whether these effects extend to subcortical auditory pathways and whether similar reweighting mechanisms operate in chronic pain populations or clinical settings involving altered sensory gating. Clarifying how pain modulates early versus later stages of sensory processing will be important for understanding the broader cognitive and functional consequences of pain on multisensory perception.

### 4.1 Limitations

The present study has two main limitations. First, because unpleasantness was assessed using a unipolar scale, the study does not address potentially pleasant aspects of non‐painful thermal stimulation and therefore cannot capture bidirectional affective responses; ratings thus reflect aversiveness within the tested range rather than general hedonic valence. Second, as in ERP research more broadly, spatial inferences are constrained by the inverse problem. Accordingly, cluster‐based permutation results are interpreted at the level of spatiotemporal clusters overlapping canonical components, rather than as precise effect onsets or offsets. Third, the ERP epochs retained for analysis included only a 50‐ms pre‐stimulus interval, which is shorter than the baseline intervals commonly recommended in ERP research (Klawohn et al. 2020). The present ERP findings should therefore be interpreted with this methodological constraint in mind.

## Author Contributions

The study was designed by all authors. Experiments and data analysis were conducted by A.L. All authors critically reviewed the results. A.L. led the preparation of the manuscript, which was subsequently edited and approved by all authors. All authors agree to be accountable for all aspects of the work.

## Funding

This work was supported by the Department of Foreign Affairs and International Trade Canada (DFAIT) through the Canadian Post‐Doctoral Research Fellowships (PDRF) awarded to A.L.; by the Natural Sciences and Engineering Research Council of Canada (NSERC) Discovery Grant awarded to M.S.; and by the Canadian Institutes of Health Research (CIHR) operating grant (MOP86541) awarded to P.R. All authors contributed to the conceptualization of the study. A.L. programmed the tasks, collected and analysed the data, and drafted the manuscript. M.S. and P.R. contributed to data analysis and provided critical revisions of the manuscript.

## Conflicts of Interest

The authors declare no conflicts of interest.

## Data Availability

The data supporting the findings of this study are available from the corresponding author upon reasonable request. All data will be shared in an anonymized format to protect participant confidentiality.
